# Experiences of cancer survivors in Europe: Has anything changed? Can artificial intelligence offer a solution?

**DOI:** 10.3389/fonc.2022.888938

**Published:** 2022-09-14

**Authors:** Iman Hesso, Reem Kayyali, Andreas Charalambous, Maria Lavdaniti, Evangelia Stalika, Maria Lelegianni, Shereen Nabhani-Gebara

**Affiliations:** ^1^ School of Life Sciences, Pharmacy and Chemistry, Kingston University London, Kingston upon Thames, United Kingdom; ^2^ Nursing Department, Cyprus University of Technology, Limassol, Cyprus; ^3^ Nursing Department, International Hellenic University, Thessaloniki, Greece; ^4^ School of medicine, Aristotle University of Thessaloniki, Thessaloniki, Greece

**Keywords:** cancer survivors, cancer care, cancer diagnosis, cancer treatment, perceptions, experiences, artificial intelligence, Europe

## Abstract

**Introduction:**

Cancer is a major global health issue. Despite technological advancements in oncology, challenges remain in many aspects related to cancer management. This study constitutes one part of the user requirement definition of INCISIVE EU H2020 project, which has been designed to explore the full potential of artificial intelligence (AI) based technologies in cancer imaging. The study aimed to explore cancer survivors’ experiences of cancer care in five European countries.

**Methods:**

A qualitative study employing semi-structured interviews was conducted. A purposive sampling strategy was used to recruit participants across the five validation countries of INCISIVE project: Greece, Cyprus, Spain, Italy, and Serbia. Forty cancer survivors were interviewed between November 2020 and March 2021. Data was analysed thematically using the framework approach and coded using NVivo12 software.

**Results:**

The analysis yielded several gaps within the cancer care pathway which reflected on the participants experiences. Five key themes were revealed; (1) perceived challenges during the cancer journey, (2) the importance of accurate and prompt diagnosis, (3) perceived need for improving cancer diagnosis, (4) absence of well-established/designated support services within the pathway and (5) suggestions to improve cancer care pathway.

**Conclusion:**

Cancer survivors experienced significant burdens pertaining to cancer diagnosis and treatment. Our findings underscored some main gaps within the cancer care pathway which contributed to the challenges articulated by the participants including lack of resources and delays in diagnostic and treatment intervals. Additionally, several suggestions were provided by the cancer survivors which could be considered towards the improvement of the current state of care, some of which can be optimised using new technologies involving AI such as the one proposed by INCISIVE.

## Introduction

Cancer is a major global health issue (1). It is a leading cause of death worldwide accounting for an estimate of 10 million deaths in 2020 (1). In Europe, 2.7 million people were diagnosed with cancer in 2020 and 1.3 million people lost their lives to it (2). This means that Europe accounts for more than a tenth of cancer deaths worldwide. Unless urgent action is taken, mortality rate is set to increase by more than 24% by 2035 in Europe (2). Hence, several policy documents in Europe are urgently calling for a renewed commitment to cancer prevention, treatment, and care through recognising the growing challenges and opportunities to overcome them. This can be done through maximising the potential of new technologies, eradication of inequalities in access to cancer knowledge, prevention, diagnosis, and care, and improving patients’ outcomes (2). One of the most important documents in this regard is the EU Beating Cancer Plan (2). The aforementioned plan aims to address the entire pathway while focusing on four key areas: prevention, early detection, diagnosis and treatment, and quality of life (2).

Cancer offers a unique context for medical decisions given not only the various disease states involved but also the need to consider the individual condition of patients, their ability to receive treatment, and their responses to treatment. Despite technological advancements in the oncology field, challenges remain in many aspects related to cancer management, mainly: accurate detection, tumor classification and characterisation, prediction of tumor evolution and precise evaluation and treatment schemas and follow-up monitoring (3). In fact, cancer burden can be significantly reduced through early detection and appropriate treatment (1). Research and innovation alongside digital technologies have radically advanced our understanding of cancer initiation, and progression, diagnosis, and prevention (2). This includes artificial intelligence (AI) which is a subfield of computer science that has been applied in medicine and in oncology specifically (4, 5). The application of AI in oncology has been rapidly emerging (6), with medical imaging being one of the focus areas (7). Nowadays AI is applied in cancer imaging including digital pathology (low- and high-level image processing and clarification tasks), radiographic imaging (differentiation between high and low risk lesions) and clinical photographs (8, 9). Medical imaging is an important part of cancer protocols applied mainly, but not limited, in diagnosis and detection stages (7). Indeed, AI promises to enhance the qualitative interpretation of cancer imaging by expert clinicians (3). Therefore, the INCISIVE project has been designed to explore the full potential of AI based solutions/technologies in cancer imaging. The project also comes in line with the current European Union (EU) policy and strategy as highlighted earlier.

INCISIVE is an EU funded project that brings together leading researchers, healthcare professionals (HCPs) and industry partners from across 9 European countries. The project aims to develop and validate an AI-based toolbox that enhances the accuracy, specificity, sensitivity, interpretability and cost-effectiveness of existing cancer imaging methods (10). INCISIVE targets the most common types of cancers: breast, prostate, lung and colorectal cancer (1, 10).

The INCISIVE project will also include an automated machine learning (ML) based annotation mechanism and the development of an interoperable pan-European federated repository of health data including medical images. The proposed repository will enable the secure donation and sharing of data in compliance with ethical, legal and privacy demands, increasing accessibility to datasets and enabling experimentation of AI-based solutions, towards the large-scale adoption of such solutions in cancer diagnosis, prediction and follow-up (10).

Care pathways for cancer management can differ between countries depending on local specificities. Hence, in the context of a collaborative EU project like INCISIVE, it is important to explore cancer survivors’ experiences and perceptions of cancer care *via* semi-structured interviews in order to get detailed accounts from participants from each country. The results of these interviews will allow the identification of the common needs, as well as the gaps and rooms for improvement regarding the current state of cancer care. Based on the generated results, the current paper will then discuss the potential of AI in cancer imaging and more specifically the technology proposed by INCISIVE in improving the cancer journey in the discussion section. This will allow for better understanding on how the INCISIVE outcomes could contribute to improving such perceptions and experiences. 

## Materials and methods

This is a qualitative study that forms a part of the user requirement definition of the INCISIVE project (https://incisive-project.eu/) (10). The research methods of this study are reported according to the Consolidated Criteria for Reporting Qualitative Research (COREQ) (11) (See **Additional File 1**).

### Study design

A qualitative approach employing semi-structured interviews was used to address the aim of this study. A semi-structured interview was used as a method since it allowed for in-depth exploration of cancer survivors’ experiences and perceptions in relation to the investigated topic. This method is underpinned by phenomenology as a philosophical approach to attain a detailed account of the phenomena under investigation from the participants’ perspective (12).

### Participants and recruitment

A purposive sampling strategy based on the knowledge of the project’s consortium was used to recruit participants across the five validation countries of INCISIVE: Greece, Cyprus, Spain, Italy, and Serbia. Eligible participants were approached by their clinicians during consultations across the participating countries. Clinicians were provided with the participants’ inclusion criteria, participant information sheet (PIS) and consent form to aid in the recruitment process. In light of the coronavirus disease-19 (COVID-19) pandemic, the clinicians provided potential participants with the PIS and consent form either *via* email in case of virtual consultations or in person in case of face-to-face consultations.

Participants were included in the study if they met the following inclusion criteria: over 18 years old, having any of the following cancer types: breast, lung, prostate or colorectal, diagnosed between 6-8 months ago, have good command of English language, capable of understanding and willing to provide voluntary informed consent. Patients were excluded if they were under 18 years of age, very recently diagnosed (under 3 months), illiterate or with language barrier, mentally incapacitated (based on medical records) or involved in another study at time of recruitment.

Sample size was determined by the concept of data saturation. Data saturation refers to the point where no new information is emerging out of the interviews. As a rule of thumb (13), the stopping criterion guiding data saturation is three, referring to the number of interviews that can be conducted without any new information, after which recruitment can be stopped. In this study saturation occurred at the 37th interview. Hence, a total of 40 participants were recruited and interviewed, and all interviews were included in the final analysis.

### Data collection

Data collection was carried out between December 2020 and March 2021. The data were collected by the first author (IH) who is a female research associate for the INCISIVE project with a Doctor of Philosophy (PhD) qualification and a considerable expertise in conducting qualitative research in healthcare. In total, 40 interviews were conducted: 32 *via* email, 3 by telephone and 5 online *via* Microsoft (MS) Teams software. Interviews conducted over the phone or *via* MS Teams lasted an average of 30 minutes (range: 25-40 mins). None of the research team had relationships with any of the participants. Hence, a brief introduction about the research was provided by IH when conducting the interviews *via* MS Teams or over the phone. In case of email interviews, a written brief introduction about the research was provided within the email sent to the participants beside the PIS that has been previously sent to them by their clinicians. Electronic informed consent was acquired from each participant before conducting the interview. No repeat interviews were conducted with any of the participants. Participants’ demographics and characteristics are summarised in **Table 1**.

**Table 1:** Characteristics of study participants (cancer survivors).

Tumor type	Number of participants
Breast cancer	17
Lung cancer	6
Colorectal cancer	10
Prostate cancer	7
**Country**	
Greece	11
Italy	9
Serbia	10
Cyprus	6
Spain	4
**Gender**	
Male	17
Female	23

### Data collection tool

The interview schedule (See **Additional File 2**) was developed by the research team to guide and facilitate data collection. The interview schedule consisted of 15 open-ended questions, covering five main sections: (1) first point of contact in the care pathway, (2) counselling and information provided during the participants journey, (3) challenges and difficulties experienced during the participants’ journey, (4) post treatment care and (5) suggestions for improving the care from the perspective of cancer survivors.

### Data analysis and reporting

Online and telephone interviews were audio-recorded and transcribed verbatim by the first author (IH). Handwritten notes were taken during online and telephone interviews. No transcription was required for interviews received *via* email. Transcripts were not returned to participants for comments. All interviews were subsequently analysed thematically using the five-stage framework approach (14–16) by the first author (IH). The first stage involved reading and re-reading a small number of the interview transcripts to achieve data familiarisation, *via* noting important patterns within the data. This enabled the identification of the initial emergent codes constituted by a collection of references indicating a pattern in how survivors experienced cancer care, challenges perceived during their journey, support received, suggestions for improvement of cancer management and any other important issues in relation to the study objectives. The second stage involved the development of the thematic framework *via* grouping the initial codes into themes and subthemes. The remaining transcripts were read and re-read to ensure that all data had been coded and the analytical framework was further developed as additional codes emerged. Derivation of themes was done using inductive/deductive approaches (from the data and literature). All themes were given an equal weighting within the thematic framework. The third stage involved numerical indexing of the developed framework by assigning numbers to the emergent themes and subtheme to apply the thematic framework to the data. Charts were then created, which summarised the views and experiences of the participants within the emergent themes and subthemes. This was followed by mapping and interpretation of the data in relation to the research objectives. The analysis process was cyclic and iterative in nature; this entailed transcribing and reading the first few interviews to achieve data familiarisation and identify preliminary codes. After that, and during the phase of data collection, each interview was transcribed (if needed) and coded to help guide with data saturation and hence recruitment. NVivo 12 software was used to facilitate data organisation and coding.

Transcript’s coding and interpretation was a continuous and extensive process; this involved discussing and checking the coding structure and the coded transcripts with the other co-authors. RK and SNG independently reviewed the coding performed by IH. Any disagreements over data coding and interpretation were discussed between IH, RK and SNG until consensus was achieved. RK has a PhD qualification and SNG has a Doctor of Pharmacy (PharmD) qualification, and both are female university academics with considerable expertise in conducting qualitative research in healthcare. The final themes and subthemes were checked and verified by all authors to ensure validity of interpretations and consistency of the findings and to overcome bias in data analysis.

The data is presented in the form of themes and subthemes which represents the findings of the whole group. Direct anonymised quotations from individual participants are used to support and validate the findings generated under each theme or subtheme. Each interviewee was assigned a pseudonym comprising of the participant number and the tumor type; for example, CS1-colorectal cancer etc. with CS standing as an acronym for cancer survivor.

### Ethical considerations

Ethical approval was granted from the Research Ethics Committee at Kingston University on 17-12-2020 (Reference No. 2714). Additionally, the following partners within the INCISIVE project also obtained ethical approvals from their corresponding institutions: Spain (Reference No. HCB/2021/055), Serbia (Reference No.4/20/2-3906], Italy (Reference No. 732/2021 and Reference No. 473/20] and Cyprus (Reference No. 2020.01.260).

## Results

The thematic analysis has illuminated 5 key themes with associated subthemes (**Table 2**).

**Table 2:** Emergent themes and subthemes from cancer survivors’ interviews.

Theme	Subtheme(s)
**Perceived challenges during the cancer journey**	Delays experienced by cancer survivors. Emotional distress endured during the diagnostic phase. Treatment burden among cancer survivors.
**The importance of accurate and prompt diagnosis for cancer survivors**	
**Perceived need for improving the accuracy of cancer diagnosis.**	
**Absence of well-established/designated support services within the official care pathway**	
**Suggestions to improve cancer pathway**	

### Perceived challenges during the cancer journey

Cancer survivors described several challenges/burdens they had to face during their diagnostic and treatment journey. Pertaining to this, the following three subthemes emerged.

#### Delays experienced by cancer survivors

Some survivors reported on the delays they experienced during the diagnostic phase and treatment as well, which was mostly noted within the public healthcare system. In some cases, this was attributed to the bureaucratic procedures within the healthcare system.

“Well part of my treatment was to have hormone injections. I had to wait, ohh I had to wait several months before the health system would pay for my radiation treatment….” (CS5-Prostate cancer)

“Another difficulty is that for each action you need a referral from your general practitioner. When you receive the referral, you schedule an examination. When you come to the general practitioner, you wait, when you come for a specialist examination, you wait again … so the patient is unfortunately forced to spend a good part of their time in the doctor’s waiting room, waiting for a referral, examination………” (CS11- Breast cancer)

“Long delay to surgery after the diagnosis, we were in a long waiting list and some delay to start chemo.” (CS17-Lung cancer)

“There were delays in my appointments with the doctors and the exams.” (CS32-Breast cancer)

Some survivors mentioned how they had to do some of the required tests and images in the private sector in order to speed up the diagnostic process and avoid further delays.

“Lot of expensive scans I have done in private clinics in order not to wait too long, to start my treatment and it is not reimbursed by insurance.” (CS30- Breast cancer)

“First, they performed the colonoscopy, and the surgeon examined me, after which I did both MRI and CT diagnostics privately to speed up the process. I didn’t want to wait for those exams…” (CS39-Colorectal cancer)

In other cases, the delay was also attributed to the COVID-19 pandemic, as highlighted in the below quotations.

“The healthcare professional team was efficient but there were delays during the various diagnostic investigations due to the COVID pandemic situation.” (CS27-Colorectal cancer)

Issues related to resources in terms of lack of manpower/staffing and equipment also contributed to the delays experienced by some survivors.

“Doctors and medical workers are overburdened due to the number of patients they see. …Apart from not having enough manpower to work with patients, I don’t think we have enough equipment needed for fast and efficient treatment and diagnosis.” (CS11- Breast cancer)

#### Emotional distress endured during the diagnostic phase

Cancer survivors expressed how they felt overwhelmed by the announcement of their diagnosis. A range of emotional responses were described by the survivors upon hearing the bad news of cancer diagnosis including feelings of fear, loss, sadness, denial, shock, confusion, hopelessness, anxiety, and stress.

“I think when you know it is stage 4 it is worrying……when you look at the statistics, I mean the statistics I have been looking at are available online anyway you know, and I know stage 4A pretty damn serious so quite stressful.” (CS6-Colorectal cancer)

“The first information about the malignancy which came from the radiologist after the ultrasound and then from the oncologist surgeon who did the biopsy shocked me and I cried during the announcement…” (CS11- Breast cancer)

Interestingly, some survivors even suggested to have a psychologist or to bring someone with them when news was broken to them of cancer diagnosis, as highlighted in the below excerpts.

“It was such a shock…. If they are going to give you bad news, they tell you to bring someone with you.” (CS3-Breast cancer)

“…. when the doctors say you are diagnosed with this kind of disease like cancer…. they should explain to you with another, umm not, it’s not a technical message…. in a simple way and be careful …… so I don’t know if they’re talking if in this moment it’s possible that there can be a psychologist next to the doctor, I don’t know, but I think the doctors they don’t learn all these things.” (CS9- Prostate cancer)

In two cases, the shock experienced upon diagnosis was attributed to the fact of misdiagnosis. The two survivors expressed how they were shocked upon receiving the right diagnosis which was after the surgery, given that all images and tests were excluding the possibility of tumour malignancy.

“In my case it was a misdiagnosis so after the surgery I came up with the right diagnosis for the first time…. I was in denial and shock with many questions and difficulty in understanding.” (CS29- Breast cancer)

Feelings of fear, pain, anxiety, stress and agony were echoed during the diagnostic phase whilst going through the various laboratory and imaging tests required to establish the final diagnosis. In some cases, respondents had to move through different hospitals and cities to perform all these tests and images which escalated the difficulties experienced in that period.

“I felt agony, stressed and anxiety.” (CS14-Lung cancer)

“CT, blood tests, MRI, scintigraphies, it was tiring I was in pain, and I had to move through hospitals, private centers, cities to do all of them.” (CS17-Lung cancer)

#### Treatment burden among cancer survivors

Some participants expressed how they were overwhelmed by the burden of treatment and how they endured psychological difficulties primarily anxiety, stress and trauma due to the side effects of the treatment.

“The chemotherapy is very difficult, the chemotherapy is difficult, I mean that was the only harm bit I would say I wouldn’t go for chemotherapy again…….” (CS6-Colorectal cancer)

“The first difficulty was psychological in nature – I lost my hair, and it was bad trauma. I lost eyelashes as well and tears kept running. I had difficulties during chemo with (drug name redacted) – I lost nails on both thumbs on my feet. This was very painful (they stayed deformed even today) and I had very good appetite. One week after the whole radio therapy treatment my skin was crackled, and the implant deformed.” (CS12- Breast cancer)

Surgical complications were also reported by some survivors, which reflected negatively on their experience.

“…. I had a pulmonologist and cardiologist cause during the surgery something went wrong but not sure what, I had an oedema on the lungs, it was something with the anesthetic I think but the doctors didn’t tell me yet what happened. So, I spent five days there in intensive care. It was bad. They affected my stomach and esophagus from the antibiotics and too much medicine in the clinic, and they also broke my nose with the COVID test …” (CS10- Breast cancer)

### The importance of accurate and prompt diagnosis for cancer survivors

Having prompt diagnosis was highlighted as an important aspect throughout some interviews in order to alleviate the anxiety and stress experienced. Another important aspect was related to the importance of establishing correct staging of cancer to assure patients’ confidence and alleviate anxiety.

“The first information about the malignancy…. shocked me and I cried during the announcement. I didn’t know which of the doctors I should contact, what to do next…… The radiologist and the surgeon were very careful and helpful seeing my reaction, they tried to provide me with a biopsy as soon as possible in order to definitely confirm the diagnosis.” (CS11- Breast cancer)

“I was confused then everything was explained to me in detail by my oncologist. I wasn’t very clear about these things after I talked to him, so he made things easier because I thought it was the end of the world but he explained that this tumour was in the very early stage….” (CS10- Breast cancer)

### Perceived need for improving the accuracy of cancer diagnosis

Some interviews also reflected the need to improve diagnosis accuracy to reduce the risk of misdiagnosis and its impact on patients’ prognosis and wellbeing as highlighted in the below excerpts:

“… it is quite a shock, you know, when you go in and you’re told, because I actually thought it was benign, all the way through I thought it was benign until the lump was removed.” (CS3- Breast cancer)

“I felt the tumour, but the mammograms were not reliable.” (CS40- Breast cancer)

### Absence of well-established/designated support services within the care pathway

As noted throughout the interviews, support received by cancer survivors was limited and was mainly within the remit of contacting their HCPs when needed for any further questions/queries and not part of designated support services that are well established or structured within the care pathway. Patients expressed that they received support from their family, friends and other patients.

“They (referring to healthcare professionals) said at any time I can contact them if I’m worried about anything or if I’m having a problem with the follow up, you know like with the medication I’m on now….” (CS3-Breast cancer)

“My family, in particular my daughter, strongly supports me.” (CS24-Colorectal cancer).

“My husband was the biggest support during all process my children and friends also, some patients who had passed the same treatment were very important source of information and the friendships.” (CS30- Breast cancer).

On the other hand, those who received some kind of support expressed their appreciation for this aspect of care.

“I received support from the urologist, for some reason his confidence about the successful outcome of the surgery filled me with optimism…” (CS1- Prostate cancer).

“I was offered to speak to a psychologist at the hospital, which I accepted, and I feel that the conversation was helpful.” (CS38- Colorectal cancer).

### Suggestions to improve cancer care pathway

A variety of recommendations were suggested by the participants throughout the interviews in an attempt to improve cancer care. Interestingly, some cancer survivors were not merely reflecting on their experiences but on those of other survivors as well when suggesting improvements for the care pathway. Some of these suggestions revolved around issues related to the healthcare system, most notably the desire for reducing waiting times for cancer diagnosis and provision of more resources mostly in terms of manpower/workforce and modern equipment, as highlighted in the below excerpts:

“In some screening departments more modern screening machines. In some screening departments there were queues and long waiting time, so more staffing therein.” (CS28- Breast cancer).

“In my opinion, in my case because I have private healthcare, so it’s no problem, it’s quite fine. But I know from colleagues who don’t have, and they passed through cancer, they had to wait a very long time. …….” (CS2-Prostate cancer).

“Widely available imaging modalities with experts available. More hospital staff, nurses especially.” (CS37-Colorectal cancer).

The interviewees also articulated other suggestions related to the healthcare system including better communication among HCPs involved in the patients’ care and having better organisation of the healthcare system itself. The below quotations provide an insight into some of these recommendations.

“And also the doctors can maybe can communicate a little bit better because you are being treated by different maybe hospitals and doctors and everything….…” (CS8- Breast cancer).

“I do realize that the staff is overworked, but the organization is bad, the people who make the decisions how to organize the system are not doctors themselves and don’t know how it should be organized.” (CS39-Colorectal cancer).

Other suggestions revolved around the approach of care itself. The need for a holistic and patient-centred approach emerged as an important issue to be addressed for promoting quality cancer care in the future. In the participants’ views, care needs to be more personalised and shaped around patients’ needs in order to improve the quality of care provided. An important aspect to this was also the provision of psychological and emotional support to patients and their families which was considered of equal importance to physical treatment.

“More use of psychological help. Holistic approach to healing and realization that maintaining the balanced psychic is as important as physical care of the body.” (CS28- Breast cancer).

Other suggestions were related to the essence of the doctor-patient relationship, as some respondents also stressed the need for more empathy, care, and trust on the part of HCPs when dealing with patients during their journey.

“Doctors are absolutely not concerned, nor are they interested in how you feel…. My general impression is that more care is taken not to make a mistake and that health protocols and bureaucratic procedures are followed, than to examine the health condition of each patient in detail and with interest ……. much more humanity on the part of doctors in dealing with oncology patients is needed” (CS11- Breast cancer).

## Discussion

The current research provides an in-depth analysis of cancer survivors’ views and experiences into the current state of cancer care and challenges encountered across several European countries mainly Greece, Italy, Cyprus, Spain and Serbia.

One of the main challenges highlighted in this study was the delays experienced during diagnosis and treatment. From cancer survivors’ perspective, these delays were related mainly to the healthcare system. In some cases, the respondents had to perform the required imaging/tests within the private healthcare system to speed up the process and avoid further delays in the diagnostic process or to travel to another city to do the imaging/tests due to unavailability in the local area. However, this speeds up the diagnostic process for the patients but at the same time it contributed potentially to financial toxicity. This in return suggests several gaps in the current state of cancer care, including the long waiting times/intervals for diagnosis and treatment in addition to the unavailability of some imaging modalities in some hospitals and specialist centres, particularly the more advanced modalities. Therefore, when cancer survivors were asked to recommend changes to the current state of care, many of the suggestions were centred on reducing waiting times and provision of more resources, particularly equipment and workforce. In fact, several studies and reviews in the literature have documented delays in cancer diagnosis and treatment due to several reasons including travel burden and transfer between hospitals, the healthcare system, in addition to clinician-related and patient-related factors (17–29). Delays in cancer diagnosis and treatment are considered as problems among healthcare system worldwide (19). Additionally, in the present study, some delays were attributed to the COVID-19 pandemic. In Europe, the impact of the COVID-19 pandemic has been severe with respect to cancer care, delaying diagnosis, disrupting prevention and treatment and affecting access to medicines (2). In fact, a decrease in the number of cancer diagnoses has been reported in Europe since the pandemic began, foreshadowing a future increase in cases (2).

Another challenge identified was the emotional distress endured by the participants during the diagnostic phase, with a lot of them expressing feelings of fear, loss, sadness, denial, shock, confusion, hopelessness, anxiety, and stress upon the announcement of diagnosis. This, in turn, highlights the need to improve this aspect of care which appears to be overlooked in the patients’ journey, given that our finding is not new in the literature (30–32). Cancer diagnosis is often traumatic, causing significant stress, anxiety and distress (32). In one research, cancer patients revealed how cancer diagnosis has evoked the feelings of uncertainty, anxiety, death, complexity, and helplessness (31). In another research which aimed at assessing patients’ perceptions of breaking bad news of cancer diagnosis, the patients revealed three main areas that were lacking in their doctors when it comes to the breaking of bad news, including (1): the need of using appropriate body language (2), management of announcement timing and (3) identifying patients’ key area of concerns (33). In fact, breaking bad news is recognised as a difficult and stressful task (34). Nevertheless, it is considered as an important component in the management of cancer patients and hence a pertinent role of HCPs (33). This provides a plausible explanation as to why even some of the participants in the current study suggested the need for a psychologist or a close person/relative when bad news is delivered. Interestingly, previous research highlighted several particular communication practices which are associated with patient satisfaction when hearing bad news of cancer diagnosis including but not limited to (1): preparation of the patient for possible cancer diagnosis and (2) availability of the people wanted by the patient to hear the diagnosis during the consultation (34).

Treatment burden was also identified as another significant challenge among cancer survivors in this study, with a lot of them highlighting the psychological impact of it. These findings are in line with previous studies (30, 35, 36). A previous study in Norway exploring HCPs’ perceptions of treatment burden among colorectal cancer patients revealed how treatment and surgical complications and side effects were considered as major treatment burden for patients that led to a prolonged hospital stay and recovery (30). Additionally, a systematic review and synthesis of qualitative research into treatment burden in lung cancer described how patients were overwhelmed by the debilitating side-effects of their treatment (36). A third study which examined the needs of patients following colorectal cancer diagnosis in England revealed how some patients experienced long term physical, social and psychological implications from their disease and treatment (35). This also alludes to another important finding in the current study which is the absence of well-established/designated support services within the care pathway across the five countries. A finding which could have potentially contributed to the negative experiences articulated by the interviewees in this study including challenges encountered during diagnosis and treatment. Whilst the importance of peer support through patient organisations cannot be denied, yet provision of adequate support from the healthcare system has been identified as a crucial aspect for amelioration of treatment burden among cancer patients (30). In the present study, the respondents identified family, friends and other patients as a main source of support, yet the presence of a proper and structured support services was viewed as a highly important aspect for improving cancer care. This also echo previous findings in the literature (37). A previous Canadian study reported that more than half of the cancer patients who had emotional concerns upon diagnosis were not referred to services that could help them with their anxieties and fears (37).

The interviews with cancer survivors also highlighted two important aspects where a technology involving AI such as the one proposed by the INCISIVE project can help. The first aspect was the importance of prompt yet accurate diagnosis for cancer survivors to alleviate anxiety and stress during the journey. The second aspect was the need among cancer survivors to improve the accuracy of cancer diagnosis and avoid instances of misdiagnosis. Pertaining to cancer imaging, AI has a great potential in aiding with the following three main clinical tasks: detection, characterization, and monitoring of tumours (3), given that assessment of imaging results most commonly relies upon visual interpretation by the expert clinicians. AI offers better recognition of complex patterns in images, thus providing the chance to transform images interpretation from a purely qualitative and subjective task to one that is quantifiable and effortlessly reproducible. Additionally, AI may quantify information from images that can go undetectable by human experts and thereby complement clinical decision making (3). As such, the INCISIVE system aims to deploy technological innovations such as AI and ML to provide better cancer detection and classification, image optimization, and clinical workflow improvement. This will be achieved by the development and validation of an AI-based toolbox that enhances the accuracy, specificity, sensitivity, interpretability and cost-effectiveness of existing cancer imaging methods. Considering the current gaps and challenges identified in this study from cancer survivors’ perspective, it becomes apparent that improving decision making with AI can be of great value for optimising and enhancing the care pathways for both clinicians and cancer survivors.

When it comes to improving the current state of cancer care, several suggestions were provided by the respondents in the current study. Suggestions for improvement focused on issues at the healthcare system-level, most notably reduction of waiting times for cancer diagnosis and treatment, provision of more modern diagnostic equipment and manpower, better communication among HCPs involved in the care of the patient and better organisation of the healthcare system suggesting some level of care fragmentation. Some suggestions were not only on a system-level but also on a practitioner-level at the same time, as some survivors stressed the need for a more holistic and personalised approach to care rather than a biomedical approach which prioritises protocols implementation and bureaucratic procedures over patients’ needs. The need for structured support was stressed in many instances during the interviews and even emerged as a theme signifying the importance and value of this aspect of care. In fact, structured support within cancer care has been reported as sparse and some of the available services can be inaccessible to many patients due to several reasons including in person delivery limiting the attendance of people in remote and rural areas, work/family responsibilities, financial stressors or mobility issues (38). A previous randomised controlled trial aimed to assess the experiences of patients with breast and colorectal cancer of navigation of cancer care services versus usual care. Interestingly, participants receiving navigation services highlighted a range of valued issues within navigation including, emotional support, assistance with information needs and problem-solving, and logistical coordination of cancer care. On the other hand, unmet cancer care needs expressed by patients randomized to usual care consisted of lack of emotional support, assistance or support with childcare, household responsibilities, and coordination of care (39). Furthermore, a recent study of breast cancer survivors’ perspectives of physical and psychological support intervention after treatment underscored how the survivors really appreciated even a simple and low-cost support program which consisted of text messaging intervention. The survivors showed great appreciation for the feelings of support and continued care and the benefits reflected by this intervention on their lifestyle during the cancer journey including exercise, diet, mental health and medication adherence (38).

### Strengths and limitations

A key strength of this study was the inclusion of participants from several European countries thus providing a more comprehensive understanding/picture of the current state of cancer care in Europe. To our knowledge, this is one of few studies that explored in detail the perceptions and experiences of survivors of the most prevalent cancer types (breast, lung, colorectal and lung cancer) from different European countries. The study had several limitations as well, which might impact the generalisability of the generated results. First, selection bias, as participation in interviews might involve the more interested and more articulate people. Second, experiences were sought among survivors with sufficient English communication skills since non-English speaking participants were excluded from the study. Third, conducting the interviews *via* videoconferencing, telephone or email may have limited some people’s ability to participate due to technology barriers (38). Fourth, few survivors were included from each of the five countries which also limits the generlisability of the study across the included countries. Nevertheless, sampling in qualitative research aims at illumination rather than representativeness.

## Conclusion

Burdens related to cancer diagnosis and treatment can be quite challenging for cancer survivors as demonstrated in the current study. Our findings highlighted some main gaps within the cancer care pathway in different European countries including lack of resources and delays in diagnostic and treatment intervals. Additionally, the current study has uncovered some aspects from cancer survivors’ perspective which can be optimised using AI technology, namely in terms of speeding up the diagnostic process and increasing diagnostic accuracy. The study has also identified several valuable suggestions from cancer survivors’ perspective that can improve and promote cancer care in the future including reduction of delays, better organisation of the healthcare system, better communication between different HCPs, provision of resources in terms of equipment and manpower, promoting a more holistic and personalised approach to care and embedding structured support services within the care pathway.

## Data availability statement

The raw data supporting the conclusions of this article will be made available by the authors, without undue reservation.

## Ethics statement

This study was reviewed and approved by the research ethics committee at Kingston University on 17-12-2020 (Reference No. 2714). Additionally, the following partners within the INCISIVE project also obtained ethical approvals from their corresponding institutions: Spain (Reference No. HCB/2021/055), Serbia (Reference No.4/20/2-3906], Italy (Reference No. 732/2021 and Reference No. 473/20] and Cyprus (Reference No. 2020.01.260). The patients/participants provided their written informed consent to participate in this study.

## Author contributions

Study was conceptualized and designed by IH, SN-G and RK. Data collection was facilitated and completed by IH. Data analysis was completed by IH, SN-G and RK. IH was responsible for drafting initial manuscript. All authors were involved in data interpretation, manuscript review, and approval of final manuscript. All authors contributed to the article and approved the submitted version.

## Funding

This work was supported by the European commission under the European Union’s Horizon 2020 research and innovation programme under grant agreement No.952179.

## Acknowledgments

The authors would like to thank all data providers within the INCISIVE consortium for their contribution to participants recruitment.

## Conflict of interest

The authors declare that the research was conducted in the absence of any commercial or financial relationships that could be construed as a potential conflict of interest.

## Publisher’s note

All claims expressed in this article are solely those of the authors and do not necessarily represent those of their affiliated organizations, or those of the publisher, the editors and the reviewers. Any product that may be evaluated in this article, or claim that may be made by its manufacturer, is not guaranteed or endorsed by the publisher.

## Abbreviations

AI, Artificial Intelligence; COREQ, Consolidated Criteria for Reporting Qualitative Research; COVID-19, Coronavirus Disease-19; CS, Cancer Survivor; EU, European Union; HCPs, Healthcare professionals; ML, Machine Learning; MS, Microsoft; PhD, Doctor of Philosophy; PIS, Participant Information Sheet; Pharm D, Doctor of Pharmacy
