# Supplementary material for: Experiences of cancer survivors in Europe: Has anything changed? Can artificial intelligence offer a solution?
Source: Front Oncol. 2022 Sep 14;12:888938. doi: 10.3389/fonc.2022.888938 (PMC9515410; doi:10.3389/fonc.2022.888938)
Supplement: Supplementary file 1 [file DataSheet_1.docx]

**Consolidated Criteria for Reporting Qualitative Research (COREQ) checklist:**

| **COREQ Criteria** | **Criteria fulfilment in the current research** |
| --- | --- |
| **Domain 1: Research team and reflexivity** | |
| **Personal Characteristics** | |
| 1. Interviewer/ Facilitator | The interviews were conducted by the first author (IH) and this was mentioned in the methodology section. |
| 2. Credentials | Credentials of the research team who conducted the analysis were mentioned in the methodology section. |
| 3. Occupation | Occupations of the research team who conducted the analysis were mentioned in the methodology section. |
| 4. Gender | The research team who conducted the analysis consisted of three female academics and this was mentioned in the methodology section. |
| 5. Experience and training | The research team who conducted the analysis have considerable experience in conducting qualitative research in healthcare settings and this was mentioned in the methodology section. |
| **Relationship with participants** | |
| 6. Relationship established | None of the research team had relationships with any of the participants prior to study commencement. |
| 7. Participant knowledge of the interviewer | There was no prior knowledge between the interviewer and the participants, that’s why a brief introduction about the research was provided by IH when conducting the interviews via Microsoft Teams or over the phone. In case of email interviews, a written brief introduction about the research was provided within the email sent to the participants beside the patient information sheetthat has been previously sent to them by their clinicians. All participants were provided by their clinicians with participant information sheet stating the details of the research. This was mentioned in the methodology section. |
| 8. Interviewer characteristics | The reported characteristics about the interviewer is the qualification, occupation, gender and that the interviewer had no previous relationships with the interviewees. This was mentioned in the methodology section. |
| **Domain 2: Study Design** | |
| **Theoretical Framework** | |
| 9. Methodological orientation and theory | The main methodological orientation was phenomenology. However, framework approach was used for data analysis. |
| **Participant selection** | |
| 10. Sampling | A purposive sampling strategy based on the knowledge of the project’s consortium (INCISIVE project) was used to recruit participants and this was reported in the methodology section. |
| 11. Method of approach | Participants were approached in person or contacted via email by their corresponding clinicians. This was reported in the methodology section. |
| 12. Sample size | 40 cancer survivors were interviewed for this research. Sample size was determined by data saturation and a stopping criterion of 3 interviews, this was mentioned in the methodology section. |
| 13. Non-participation | All participants approached accepted to participate as indicated by the consortium partners who recruited cancer survivors. Hence, there was no mention about non-participation in the article. |
| **Setting** | |
| 14. Setting of data collection | The interviews were conducted either via email, over the phone or virtually via Microsoft Teams. This was mentioned in the methodology section. |
| 15. Presence of non-participants | The interviews were conducted either via email, over the phone or virtually via Microsoft Teams so there was no presence of non-participants during the interviews. |
| 16. Description of sample | Basic characteristics about the interviewees such as gender, country of residence, and disease condition (tumour type) were provided in the methodology section in table 2. |
| **Data Collection** | |
| 17. Interview guide | A copy of the interview guide was provided as an additional file (Please refer to additional file 2). |
| 18. Repeat interviews | No repeat interviews were conducted, and this was mentioned in the methodology section. |
| 19. Audio/ visual recording | All interviews that were done via Microsoft Teams or over the phone were audio-recorded. |
| 20. Field notes | Handwritten notes were taken during the online interviews and interviews conducted over the phone. |
| 21. Duration | Duration of the online and telephone interviews was mentioned in the methodology section. |
| 22. Data saturation | Sample size was guided by data saturation, and this was reported in the methodology section. |
| 23. Transcripts returned | Transcripts were not returned for participants to check and comment. |
| **Domain 3: analysis and findings** | |
| **Data analysis** | |
| 24. Numbers of data coders | Data coding was done by the first author. However, two authors independently reviewed the coding done by the first author and discussions were taking place in case of disagreement. The coding framework was extensively discussed and agreed by all authors. |
| 25. Description of the coding tree | Description of the coding tree/ framework was provided, please refer to the methodology section. |
| 26. Derivation of themes | Derivation of themes was done using inductive/ deductive approaches i.e.: from the data and literature review. |
| 27. Software | Management and coding of the data was done using NVivo 12 software. |
| 28. Participant checking | This was not conducted. |
| **Reporting** | |
| 29. Quotations presented | Participants’ quotations were included in the results section, and this was mentioned in the methodology section. |
| 30. Data and findings consistent | The final themes and subthemes were checked and verified by all authors to ensure validity of interpretations and consistency of the findings. |
| 31. Clarity of major themes | All themes were given an equal weighting within the thematic framework. |
| 32. Clarity of minor themes | All themes were given an equal weighting within the thematic framework. |
