# Supplementary material for: Experiences of cancer survivors in Europe: Has anything changed? Can artificial intelligence offer a solution?
Source: Front Oncol. 2022 Sep 14;12:888938. doi: 10.3389/fonc.2022.888938 (PMC9515410; doi:10.3389/fonc.2022.888938)
Supplement: Supplementary file 2 [file DataSheet_2.docx]

Interview schedule for cancer survivors

**Section1: The care pathway in general and first point of contact**

1- How do you feel about the cancer care that you are receiving?

Prompt: enquire more about the causes of their feeling(s), so why they were for example dissatisfied, satisfied, neutral etc… depending on the answer.

2- Who was the first point of contact in that journey? (the first healthcare professional that you have seen)

3- How many healthcare professionals were involved in your care? Can you list them?

**Section 2: Counseling and information**

4- What information was given to you when you were first diagnosed?

Prompt: ask participants about the information content: information about the condition, diagnosis options, and treatment options.

Prompt: ask participants about the type of the information whether verbal, written (e.g: leaflets), written and verbal or online.

5- Who provided you with this information?

6- How do you feel about the information given to you about your condition?

Prompts:

- Was the information easy to understand or not? Why?
- Was the information useful and helpful or not? Why?
- Was the information enough or not? Why?

**Section 3: Challenges and difficulties including delays**

- **Diagnosis**

7- How many times did you have to visit the gatekeeper/the GP before they had a suspicion about your case/ they referred you to screening?

Prompt: Also enquire if they referred themselves?

8- How many visits did you have before you had your final diagnosis (i.e. the clinician/the GP told you about your condition)?

9- What were the various tests and imaging scans that you had, and how did you feel about the whole process?

10- What are the difficulties that you have faced, if any, during diagnosis?

Prompt: Ask participants if they had any problems or delays in doing the necessary tests and images, and let them explain in more details.

- **Treatment**

11- What are the difficulties that you have faced, if any, during your treatment?

Prompt: Ask participants if they had any problems or delays in receiving/having their cancer treatment/surgery and let them explain in more details.

**Section 4: Monitoring and follow-up**

12- What kind of support have you been receiving during your journey?

Prompts:

- Who provided you with this support?
- Do you feel you had enough support? Why?

13- What kind of support are you now receiving/getting?

Prompts:

- Who is providing you with this support?
- Do you feel you are having enough support? Why?

14- What is the follow-up programme / scheme that you are having now?

Prompt: Enquire about the frequency of follow-up (how often): do you have check-up every 3 months, 6 months or longer duration?

Prompt: Enquire about the follow-up tests done each time/visit (type): blood checks, imaging tests etc……

**Section 5: Suggestions for improving the care pathway:**

15- What changes would you like to see in the future about cancer care in your country?

**Further additions/ comments**

Do you have any questions that you would like to ask me?

Is there anything else you would like to share or add about this subject?
